# Effect of index HIV self-testing for sexual partners of clients enrolled in antiretroviral therapy (ART) programs in Malawi: A randomized controlled trial

**DOI:** 10.1371/journal.pmed.1004270

**Published:** 2023-08-04

**Authors:** Kathryn Dovel, Kelvin Balakasi, Khumbo Phiri, Frackson Shaba, Ogechukwu Agatha Offorjebe, Sundeep K. Gupta, Vincent Wong, Eric Lungu, Brooke E. Nichols, Tobias Masina, Anteneh Worku, Risa Hoffman, Mike Nyirenda

**Affiliations:** 1 Division of Infectious Diseases, David Geffen School of Medicine, University of California, Los Angeles, California, United States of America; 2 Partners in Hope, Lilongwe, Malawi; 3 David Geffen School of Medicine, University of California, Los Angeles, California, United States of America; 4 School of Medicine, Charles R. Drew University of Medicine and Science, Los Angeles, California, United States of America; 5 USAID Global Health Bureau, Arlington, Virginia, United States of America; 6 Health Economics and Epidemiology Research Office, Department of Internal Medicine, School of Clinical Medicine, Faculty of Health Sciences, University of the Witwatersrand, Johannesburg, South Africa; 7 Department of Global Health, School of Public Health, Boston University, Boston, Massachusetts, United States of America; 8 Malawi Ministry of Health, HIV/AIDS Unit, Lilongwe, Malawi; 9 USAID Global Health Bureau, Lilongwe, Malawi

## Abstract

**Background:**

HIV testing among the sexual partners of HIV–positive clients is critical for case identification and reduced transmission in southern and eastern Africa. HIV self-testing (HIVST) may improve uptake of HIV services among sexual partners of antiretroviral therapy (ART) clients, but the impact of HIVST on partner testing and subsequent ART initiation remains unclear.

**Methods and findings:**

We conducted an individually randomized, unblinded trial to assess if an index HIVST intervention targeting the partners of ART clients improves uptake of testing and treatment services in Malawi. The trial was conducted at 3 high-burden facilities in central and southern Malawi. ART clients attending HIV treatment clinics were randomized using simple randomization 1:2·5 to: (1) standard partner referral slip (PRS) whereby ART clients were given facility referral slips to distribute to their primary sexual partners; or (2) index HIVST whereby ART clients were given HIVST kits + HIVST instructions and facility referral slips to distribute to their primary sexual partners. Inclusion criteria for ART clients were: ≥15 years of age, primary partner with unknown HIV status, no history of interpersonal violence (IPV) with partner, and partner lives in facility catchment area. The primary outcome was partner testing 4-weeks after enrollment, reported by ART clients using endline surveys. Medical chart reviews and tracing activities with partners with a reactive HIV test measured ART initiation at 12 months. Analyses were conducted based on modified intention-to-treat principles, whereby we excluded individuals who did not have complete endline data (i.e., were loss to follow up from the study). Adjusted models controlled for the effects of age and marital status.

A total of 4,237 ART clients were screened and 484 were eligible and enrolled (77% female) between March 28, 2018 and January 5, 2020. A total of 365 participants completed an endline survey (257/34 index HIVST arm; 107/13 PRS arm) and were included in the final analysis (78% female). Testing coverage among sexual partners was 71% (183/257) in the index HIVST arm and 25% (27/107) in the PRS arm (aRR: 2·77, 95% CI [2·56 to 3·00], *p* ≤ 0.001). Reported HIV positivity rates did not significantly differ by arm (16% (30/183) in HIVST versus 15% (4/27) in PRS; *p* = 0.99). ART initiation at 12 months was 47% (14/30) in HIVST versus 75% (3/4) in PRS arms; however, index HIVST still resulted in a 94% increase in the proportion of all partners initiating ART due to higher HIV testing rates in the HIVST arm (5% partners initiated ART in HVIST versus 3% in PRS). Adverse events including IPV and termination of the relationship did not vary by arm (IPV: 3/257 index HIVST versus 4/10 PRS; *p* = 0.57). Limitations include reliance on secondary report by ART clients, potential social desirability bias, and not powered for sex disaggregated analyses.

**Conclusions:**

Index HIVST significantly increased HIV testing and the absolute number of partners initiating ART in Malawi, without increased risk of adverse events. Additional research is needed to improve linkage to HIV treatment services after HIVST use.

**Trial registration:**

ClinicalTrials.gov, NCT03271307, and Pan African Clinical Trials, PACTR201711002697316.

## Author summary

**Why was this study done?:**

- Undiagnosed and untreated HIV remains a barrier to sustained epidemic control, particularly among men.
- HIV self-testing (HIVST) improves HIV testing coverage and can be distributed through sexual networks of antiretroviral therapy (ART) clients (i.e., index HIVST) who are at increased risk of HIV infection.
- A key innovation for this study is to understand the acceptability and impact of index HIVST among sexual partners of index ART clients and ART initiation rates among partners with reactive HIVST results.

**What did the researchers do and find?:**

- We conducted an individually randomized, unblinded trial with 365 participants in Malawi (*n* = 257 index HIVST arm; *n* = 107 partner referral slip (PRS) arm) to assess the impact of index HIVST on HIV testing and uptake of ART among partners of ART clients.
- Index HIVST increased HIV testing among partners (71% with index HIVST versus 25% with standard of care), was cost-efficient, and was acceptable and feasible to both ART clients and their partners.
- ART initiation among those with a reactive HIV test was lower in the index HIVST arm compared to standard of care (47% versus 75%), but the proportion of all partners who initiated ART was higher because testing uptake was substantially higher in the index HIVST arm (5% versus 3% of all partners).

**What do these findings mean?:**

- Index HIVST is a useful tool to increase HIV testing among sexual partners of ART clients in Malawi, where the majority ART clients were married and already disclosed their HIV status to their partners.
- Index HIVST may be most successful for treatment uptake when implemented alongside other strategies to increase access and demand for treatment services.
- Our sample size was not powered on secondary outcomes and did not allow for disagregatted findings by key variables such as relationship status or status disclosure. Additional research is needed to assess if index HIVST is acceptable and impactful in settings with less stable relationships and lower rates of status disclosure.

## Abstract 3

In a randomized controlled trial from Malawi, Kathryn Dovel and colleagues, investigate the impact of HIV self-testing in partners of people taking antiretroviral therapy.

## Introduction

Index partner testing, whereby the sexual partners of individuals living with HIV are tested for HIV, is critical to reaching the first 95 of the UNAIDS 95-95-95 goals (i.e., 95% of individuals living with HIV know their status) [1]. Index partner testing is associated with higher testing yield compared to other case-finding strategies [2–4] and high rates of status disclosure among partners, which can promote antiretroviral therapy (ART) adherence [5–7]. Index partner testing is also critical for identifying individuals who are HIV–negative and can benefit from HIV prevention measures [4,8,9]. However, current index testing strategies have limited reach. In Malawi, standard of care referrals (usually paper forms given for partners) result in only 22% of partners tested for HIV [10]. Male partners are least likely to test. Increasing testing among male partners, and linkage to care, promises to have massive impacts on reduced HIV transmission among women and young girls [11]. Voluntary assisted partner disclosure and testing, whereby health care workers (HCWs) visit index partners in the community to provide HIV testing services, promises to increase testing uptake [12], but is costly [13] and has increased risk for coercion and unwanted disclosure for the ART client, especially among women living with HIV [14–17]. New strategies are needed to effectively reach partners with index testing, particularly men.

HIV self-testing (HIVST) offers an alternative strategy to improving index partner testing by allowing people living with HIV to take an HIVST kit home to their sexual partner (“index HIVST”). HIVST can be used in the privacy of partners’ own homes and at times convenient to them [9] and can largely address barriers to standard index partner testing, including distance to the health facility, time associated with seeking HIV testing, and concerns related to privacy of HIV testing and unwanted disclosure [18]. Oral-based HIVST has been widely used throughout sub-Saharan Africa and has been shown to be highly acceptable among hard-to-reach populations, including men [19,20].

A growing body of literature shows that women seeking antenatal (ANC) services and female sex workers are willing and able to distribute HIVST to their male partners [21–23], with 79% to 91% of male partners reported as using an HIVST kit with minimal adverse events [24]. Two studies to our knowledge have examined the impact of secondary distribution of HIVST kits for partners of individuals living with HIV and have found index HIVST to be feasible and acceptable [25,26]; however, there is little data about treatment initiation overtime for those who use HIVST. Further, there is very little data about feasibility and acceptability from the partner perspective. Acceptability and feasibility of index HIVST, as well as linkage to additional HIV services, may differ substantially among individuals living with HIV as ART clients may fear unwanted status disclosure to their sexual partner and may be at higher risk of adverse events, such as interpersonal violence (IPV) and/or termination of a relationship.

Further, the barriers to and frequency of partner confirmatory testing and ART initiation after receiving a positive HIVST result at home are still unclear [18]. Reported ART initiation rates among HIVST users vary between 23% and 68% [27–30]. Partners of individuals living with HIV may be more likely to initiate ART as compared to the general population given that they may already be exposed to ART services (if their partner is actively in care) and have immediate social support for navigating ART clinics, although this has not been examined to our knowledge.

We conducted an individually randomized controlled trial (RCT) in Malawi to assess the impact of index HIVST on testing uptake among ART clients’ primary sexual partners as compared to standard of care partner referral slips (PRS) and describe ART initiation among diagnosed individuals.

## Methods

### Ethics statement

This study was approved by the National Health and Sciences Research Committee in Malawi and the Institutional Review Board at the University of California Los Angeles, Los Angeles (California, United States of America) (Protocol #1664). Individuals who provided oral consent for screening were screened and, if eligible, completed written informed consent for study participation.

We conducted an individually randomized, unblinded trial among ART clients and their partners in Malawi between March 28, 2018 and January 5, 2020. The primary outcome was partner status ascertainment 4 weeks after enrollment, measured through secondary reports by ART clients. Secondary outcomes included: HIV positivity rates, ART initiation among those reported as having a reactive test, usability and acceptability of index partner HIVST, and presence of adverse events, defined as self-reported presence of IPV and/or termination of romantic relationship. This study is reported as per the Consolidated Standards of Reporting Trials (CONSORT) guideline (see S1 CONSORT Checklist.). A full study protocol can be found here (see S1 Text). The trial is registered with ClinicalTrials.gov, NCT03271307 and Pan African Clinical Trials, PACTR201711002697316.

### Study sites and setting

A convenience sample of 3 high-burden referral hospitals in central and southern Malawi (Chikwawa, Nsanje, and Lilongwe districts) were included in the trial. At the time of the study, the national HIV prevalence was 9·6% [31], and 36% to 49% of adults in Malawi had been tested for HIV in the prior year, with Malawi national guidelines stipulating that index testing should be offered to all partners of clients living with HIV [32].

### Study population

Individuals living with HIV and on ART were recruited during routine ART clinic visits. Eligibility criteria included: ≥15 years of age; have a sexual partner in the last 12 months with an unknown HIV status, defined as never tested HIV–positive or not tested HIV–negative within the past 3 months (if multiple partners, individuals were asked to define the HIV status of their primary partner); no history of IPV with that partner; and partner lives within the facility catchment area.

### Randomization and masking

Computer-generated simple randomization was used to assign clients to either the PRS or HIVST arms in a ratio of 1:2·5, respectively. We intentionally randomized more clients to the HIVST arm to examine associations between key client–partner relationship dynamics and implementation outcomes in the treatment group (such as acceptability and feasibility of HIVST distribution, HIVST use and any changes to relationship dynamics, not included in this paper). Relationship dynamics of most interest include: relationship type (i.e., married or cohabitating), HIV status disclosure (or not), and decision-making dynamics within the relationship. The secondary analysis is important to identify if unique implementation strategies should be considered for different relationship dynamics, should the intervention be successful. This was a non-blinded trial therefore masking was not included.

### Description of intervention arms

#### Standard of care PRS

ART clients were counseled on the importance of index HIV testing and strategies for disclosure and offered a PRS slip and linkage card with a map of the facility to give to their primary partner.

#### Index HIVST

ART clients were counseled on the importance of index HIV testing and strategies for disclosure, given a 10-min demonstration on how to use the HIVST kit, given 1 Oraquick oral HIVST kit for their primary sexual partner, an instructional leaflet on how to use HIVST, and a linkage card with a map to the health facility. To allow for the possibility that partners may be uncomfortable with HIVST, ART clients in the HIVST arm were also given a PRS so that a partner could opt out of HIVST and test via standard HIV testing methods at the nearest health facility. Clients who were confirmed HIV–positive were referred to the facility’s ART clinic for same-day ART initiation.

### Study procedures

#### Recruitment and enrollment

ART clients were recruited by study staff while waiting for routine ART services. Individuals who provided oral consent for screening were screened in a private location within the health facility and, if eligible, completed written informed consent. Immediately following randomization, the intervention (PRS or index HIVST) was delivered by a trained study staff member.

#### Surveys with ART clients

ART clients completed a baseline survey immediately after receiving the intervention (PRS or index HIVST). Baseline survey included the following domains: (1) sociodemographics of the ART client and partner; (2) history of HIV service utilization; and (3) relationship characteristics with their primary partner.

ART clients were scheduled for an endline survey 4 weeks after study enrollment, at the same health facility to assess primary and secondary outcomes. Endline surveys included the following domains: (1) distribution and acceptability of the intervention (PRS or HIVST); (2) index partners use of HIV services since enrollment; and (3) any adverse events experienced since enrollment, such as IPV or termination of the sexual relationship. Both baseline and endline surveys lasted approximately 60 min and were conducted in the local language (Chichewa) by trained study staff. Identifiers were collected for index partners who were reported to have a positive HIV test during the study period (i.e., name, age, address, ART number—if any).

#### Medical chart reviews

Study staff conducted medical chart reviews at all PEPFAR-supported facilities in the study districts at 3, 6, and 12 months post enrollment to assess if index partners initiated ART. At the end of 12 months, those with positive HIV tests who were not found in medical chart reviews were contacted by facility staff (via phone or in person) to ascertain ART initiation outcomes, per standard of care facility protocols.

#### Surveys with partners in index HIVST arm

In the HIVST arm, we also recruited a subset of index partners to complete a survey regarding index HIVST feasibility and acceptability. ART clients in the HIVST arm were given a study recruitment card requesting their partner to present at the health facility for a study survey approximately 4 weeks after the ART client enrolled in the study. Eligibility criteria were: ≥15 years of age, was a sexual partner of a study ART client, was given a HIVST kit by a study ART client, and presented to study staff at participating facilities. Surveys were conducted in the local language (Chichewa) by a trained study staff member in a private space at the facility and lasted approximately 1 h. Survey domains included: experience with HIVST and other HIV services in the past 4 weeks, history with HIV services prior to 4 weeks ago, acceptability of index HIVST, and challenges experienced with index HIVST.

### Study outcomes

The protocol-defined primary outcome was the proportion of index partners who tested for HIV within 4 weeks after enrollment of the ART client (including either HIVST or standard blood-based testing) and was measured by secondary report from the client. ART clients completed endline surveys between 4 and 6 weeks after enrollment.

Prespecified secondary outcomes included: (1) proportion of ART clients who reported distributing the intervention to their partner; (2) reported HIV positivity rate among partners tested; (3) ART initiation within 12 months after receiving a reactive blood- or self-test (using medical charts); (4) acceptability and usability of the intervention (PRS or HIVST) from the perspective of the ART client (variables measured on a 4-point Likert scale and dichotomized for analysis as agree/strongly agree or disagree/strongly disagree); (5) presence of adverse events for the ART client. We define the presence of an adverse event if one of the 2 following categories was reported during the study period: (1) IPV (measured using questions adapted from the Malawi Demographic and Health Survey) [33]; or (2) termination of the relationship during the study period; and (6) cost. Exploratory secondary outcomes included: (1) HIV positivity rate among all partners; (2) ART initiation within 12 months among all partners; and acceptability and usability of index HIVST from the perspective of sexual partners.

### Statistical analysis

The statistical power was estimated for the primary outcome (partner uptake of HIV testing within 4 weeks after ART client enrolled in the study). With a type I error of 0.05 and sample sizes of 110 in standard of care and 250 in HIVST arms (randomized 1:2·5), we had >90% power to detect a difference in partner testing coverage of 40% in standard of care and 60% in HIVST. We inflated the sample size to account for potential loss to follow-up, assuming that 20% of individuals will be lost to follow-up (LTFU) in either arm and 15% have missing data. We use conservative outcome estimates based on preliminary data.

We used the CONSORT standards for reporting trial outcomes. All analyses were prespecified based on the protocol. Descriptive statistics (mean, standard deviation, median, inter-quartile range, and frequency distribution) were generated for all demographic and clinical information to characterize the study population overall and by study arm. We used a modified intention-to-treat analysis whereby ART clients who were LTFU (i.e., could not be contacted to complete an endline survey) were excluded from the analysis since it was impossible to determine outcomes. Clients who participated in the endline survey but had missing data for the primary outcome (10%; 35/364), or were unsure of primary outcome (2%; 9/364), were counted as failures (i.e., no HIV test completed by partner). We conducted sensitivity analyses using true intention-to-treat principles whereby those LTFU for endline surveys were counted as failures—analyses showed similar findings as the modified intention to treat approach (see S1 Table). Univariate and multivariate binomial regressions were used to compare primary outcomes between arms (we fitted a binomial model because the outcome was common). Adjusted models controlled for the effects of age and marital status as these covariates are highly associated with HIV testing and treatment outcomes in the literature. We conducted sensitivity analyses accounting for other covariates—including additional covariates did not meaningfully change the relationship between intervention and outcomes (see S2 Table). Secondary outcomes such as HIV-positivity rates, ART initiation, intervention acceptability, and presence of adverse events were described using descriptive statistics and were explored using univariate and multivariate binomial regressions, when sample size allowed. We completed subgroup analyses by sex to assess primary and secondary outcomes for men and women separately. Analyses were done with R version 3.5.3.

### Cost outcomes and analysis

Total cost per individual with a reported positive test result and per individual linked to care were calculated using study data for both study arms. Costs were derived from a recent facility-based HIVST trial in which the cost of facility-based testing and HIVST were collected at neighboring sites in Malawi [27].

In short, we applied a microcosting (bottom-up) approach from providers’ perspective, using the HIV Counselling and Testing costing tool developed by the Health Economics and Epidemiology Research Office in South Africa [34]. Data were collected from 5 facilities. Resource costs included testing consumables and equipment, staff costs, staff training, shared costs (e.g., cleaning, stock taking, data capturing, HIV counseling, and testing staff scheduling), and overhead costs (including building maintenance and utilities) and we averaged costs across all 5 facilities and reported SDs around these costs. The cost of ART initiation itself was not included in the final production cost or the total cost to initiate one new client on ART. Total testing-related costs per test completed, per newly diagnosed HIV–positive individual, and per person initiating ART were calculated by study group. All costs are reported in 2017 US$. See parent paper for more details [27].

For the PRS arm, the costs included: cost of counseling when given the PRS, cost of the partner testing at the clinic for those who returned, and the cost of confirmatory testing for those who tested positive. For the index HIVST arm, the costs included: cost of counseling when the HIVST kits were distributed, the cost of the distributed self-tests, and the cost of the clinic-based testing algorithm following a positive HIVST. For each cost component, the cost of human resources, training, equipment, consumables, and overhead were included. Costs are reported in 2018USD to reflect the time period of this trial. The costs of human resources, training, equipment, and overhead were inflated to 2018USD, while the cost of human resources, equipment, and overhead were adjusted from 2017USD to 2018USD to reflect the time period of this trial. Cost of HIV testing kits (both HIVST and provider testing kits) did not change between 2017 and 2018. The cost of an HIVST kit was assumed to be $2.

Three types of cost outcomes were assessed: (1) Cost per test provided: calculated as the cost per person counseled and given PRS or HIVST; (2) cost per person aware of HIV status: calculated as the total cost of providing PRS or HIVST to all individuals in the trial divided by the number of people newly aware of their HIV–positive status (by arm); (3) cost per ART initiate: calculated as the total cost of providing PRS or HIVST to all individuals in the trial divided by the number of people newly initiating on ART (by arm).

## Results

**Fig 1:**
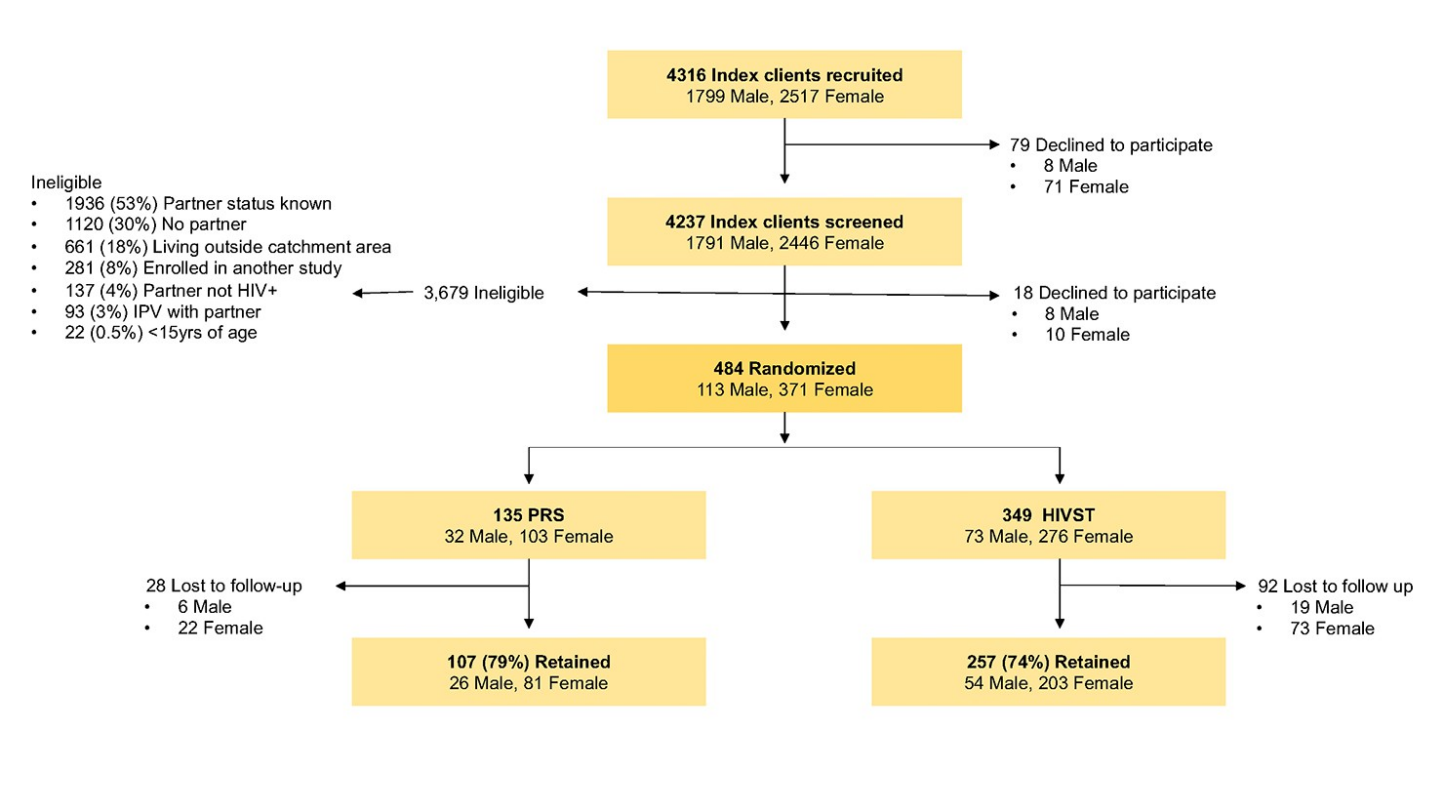
Recruitment and enrollment of ART clients. ART, antiretroviral therapy; HIVST, HIV self-test; IPV, interpersonal violence; PRS, partner referral slip.

A total of 4,237 ART clients (1,791 men and 2,446 women) were screened. Fig 1 shows the recruitment and enrollment of ART clients. Of those approached for screening, 18 declined to participate and 3,679 (91%) did not meet eligibility criteria. The primary reasons for exclusion were partner had a known HIV status (53%) and ART client did not have a sexual partner at the time of recruitment (30%). A total of 484 ART clients were enrolled (349 randomized to the index HIVST arm and 135 to the PRS arm). Of the 484,364 clients completed an endline survey at 4 weeks after enrollment (mean length: 6 weeks; SD: 2·9 weeks; range: 4·3 to 7·4 weeks), with similar retention rates by arm (79% (107/135) ART clients retained in PRS arm; 74% (257/349) retained in HIVST arm). Primary outcome results are reported for the 364 ART clients (with secondary reports for their partners).

Table 1 shows characteristics of ART clients and their index partners enrolled in the trial (*n* = 484). Most clients were female 77% (371/484), mean age was 37 years (SD 11.4 years), and only 53% (258/484) had worked for pay in the past month. Nearly 83% (402/484) of ART clients were married or living with their index partner, with a median relationship length of 5 years (IQR 2 to 14), and 66% (304/484) with at least 1 living child with their primary partner. Most ART clients (91%; 422/484) had disclosed their HIV status to their index partner prior to enrolling in the trial and talked with this partner at least once per week (96%, 443/484). Demographics were similar across arms. S3 describes characteristics of ART clients and index partners among those with a primary outcome (*n* = 364) and shows similar baseline characteristics as described in Table 1.

**Table 1:** Baseline characteristics, reported by ART client (*n* = 484).

Respondent characteristic	Total	PRS arm	HIVST arm
	*n* (%)	*n* (%)	*n* (%)
n	484	135 (27.9%)	349 (72.1%)
**Client information**			
**Demographic variables**			
Male	113 (23.4%)	30 (22.2%)	83 (23.8%)
Mean age, years (SD)	37 (11.4)	37 (12.0)	37 (11.2)
Median years of education, (IQR)	5 (1–8)	4 (1–8)	5 (1–8)
Relationship type with primary partner			
Married/living together	402 (83.1%)	112 (83.0%)	290 (83.1%)
Steady partner	56 (11.6%)	19 (14.1%)	37 (10.6%)
New/infrequent partner	26 (5.4%)	4 (2.9%)	22 (6.3)%)
Median years in a relationship, (IQR)	5 (2–14)	4 (2–15)	6 (2–13)
Household wealth quantiles			
Low quantile	163 (33.7%)	51 (37.8%)	112 (32.1%)
Middle quantile	160 (33.0%)	35 (25.9%)	125 (35.8%)
High quantile	161 (33.3%)	49 (36.3%)	112 (32.1%)
Worked for pay in last month	258 (53.3%)	67 (49.6%)	191 (54.7%)
Median number of living children, (IQR)	3 (2–5)	3 (2–5)	3 (2–5)
Has living child with the primary partner	304 (65.8%)	79 (61.7%)	225 (67.4%)
**HIV services**			
Median years on ART, (IQR)	5 (1.6–7.8)	5 (1.3–7.9)	4 (1.6–7.7)
Disclosed HIV status to a non-sexual partner	422 (87.2)	119 (88.1)	303 (86.8)
Have a close friend on ART (talk with them at least 1/week)	220 (53.8%)	63 (53.4%)	157 (54.0%)
More than 1 sexual partner in past 12 months	40 (8.3%)	13 (9.6%)	27 (7.7%)
**Primary partner information**			
**Demographic variables**			
Male	371 (76.7%)	105 (77.8%)	266 (76.2%)
Mean age, years (SD)	39 (11.8)	40 (13.2)	39 (11.1)
**Relationship with primary partner**			
Disclosed HIV status to primary partner	422 (91.3%)	115 (89.8%)	307 (91.9%)
Talk with partner at least once per week	443 (95.9%)	123 (96.1%)	320 (95.8%)
Confident they would still be with this partner in 12 months	387 (83.8%)	103 (80.5%)	284 (85.0%)
**Region**			
Southern	283 (58.5%)	84 (62.2%)	199 (57.0%)
Central	201 (41.5%)	51 (37.8%)	150 (43.0%)

ART, antiretroviral therapy; HIVST, HIV self-test; PRS, partner referral slip.

### Primary outcomes

Reported HIV testing among index partners within 4 weeks of ART client enrollment was significantly higher in the HIVST arm as compared to the PRS arm (71% [183/257] versus 25% [27/107], respectively; aRR: 2·77, 95% CI [2.56 to 3.00], *p* ≤ 0.001). Stratified analyses by sex show that testing coverage increased with index HIVST relative to PRS by over 2-folds (aRR 2·38, 95% CI [1·86 to 3·06], *p* ≤ 0.001) among male index partners and by 3-fold (aRR 3·19, 95% CI [2·68 to 3·81], *p* ≤ 0.001) in female index partners (see Table 2).

**Table 2:** HIV testing uptake among partners, reported by ART clients (*n* = 364).

HIV testing service	PRS arm	HIVST arm	RR (95% CI)	*p*-Value	aRR (95% CI)^	*p*-Value
*n* (%)	*n* (%)
ART client delivered intervention	98/107 (91.6%)	231/257 (89.9%)	0.98 (0.89, 1.08)	0.712	0.97 (0.90, 1.04)	0.414
Female index partner	73/81 (90.1%)	179/203 (88.2%)	0.98 (0.84, 1.13)	0.771	0.99 (0.88, 1.11)	0.829
Male index partner	25/26 (96.2%)	52/54 (96.3%)	1.00 (0.93, 1.08)	0.968	0.93 (0.90, 0.96)	<0.001
Index partner tested	27/107 (25.2%)	183/257 (71.2%)	2.82 (2.64, 3.02)	<0.001	2.77 (2.56, 3.00)	<0.001
Female index partner	17/81 (21.0%)	135/203 (66.5%)	3.17 (2.79, 3.60)	<0.001	3.19 (2.68, 3.81)	<0.001
Male index partner	10/26 (38.5%)	48/54 (88.9%)	2.31 (1.81, 2.95)	<0.001	2.38 (1.86, 3.06)	<0.001
Index partner tested HIV–positive	4/27 (14.8%)	30/183 (16.4%)	1.11 (0.43, 2.85)	0.834	0.82 (0.28, 2.35)	0.707
Female index partner	4/17 (23.5%)	28/135 (20.7%)	0.88 (0.37, 2.09)	0.775	0.78 (0.32, 1.95)	0.601
Male index partner	0/10 (0.0%)	2/48 (4.2%)	-	-	-	-
HIV+ index partner-initiated ART at 12 months	3/4 (75.0%)	14/30 (46.7%)	-	-	-	-
Female index partner	3/4 (75.0%)	14/28 (50.0%)	-	-	-	-
Male index partner	0 (0.0%)	0/2 (0.0%)	-	-	-	-

^^^Adjusted for age and marital status.

aRR, adjusted rate ratio; ART, antiretroviral therapy; CI, confidence interval; HIVST, HIV self-test; PRS, partner referral slip; RR, rate ratio.

Reported HIV testing among index partners within 4 weeks of ART client enrollment was significantly higher in the HIVST arm as compared to the PRS arm (71% [183/257] versus 25% [27/107], respectively; aRR: 2·77, 95% CI [2 56 to 3·00], *p* ≤ 0.001). Stratified analyses by sex show that testing coverage increased with index HIVST relative to PRS by over 2-folds (aRR 2·38, 95% CI [1·86 to 3·06], *p* ≤ 0.001) among male index partners and by 3-fold (aRR 3·19, 95% CI [2·68 to 3·81], *p* ≤ 0.001) in female index partners (see Table 2).

### Secondary outcomes

#### Distribution of intervention to index partner

The majority of ART clients in both arms reported having distributed the intervention to their index partner (92% for PRS [98/107] and 90% for self-test [231/257]; *p* = 0.615) (Table 2). Reasons for not delivering either PRS or HIVST were similar across arms: 12/35 (34%) had not seen their partner since enrolling in the study, 11/35 (31%) had not disclosed their HIV status, 6/35 (17%) knew that their partners were already HIV–positive, and 11% (4/35) were afraid of their partner’s response.

### Reported HIV positivity rate

Reported HIV-positivity among index partners who tested was similar across arms, with 15% (4/27) testing positive in the PRS arm and 16% (30/183) in the HIVST arm (Table 2). While positivity rates were similar across arms (*p* = 0.834), high testing coverage among HIVST partners translated to a greater proportion of partners identified as HIV–positive (11%; 30/257) as compared to PRS (4%; 4/107).

### ART initiation

All index partners in the PRS arm who tested HIV–positive (*n* = 4) tested at a health facility using standard blood-based testing administered by an HCW, and 3/4 (75%) initiated ART that same day, likely facilitated by facility HCWs. In the HIVST arm, 47% (14/30) of index partners who tested HIV–positive were identified as having initiated ART within 12 months of their test. The vast majority doing so within 3 months after being given an HIVST kit (93%, 13/14) (S1 Fig).

### Acceptability and adverse events

Table 3 shows intervention acceptability and adverse events outcomes as reported by ART clients. Over 98% (325/329) of ART clients in both arms were comfortable explaining the respective intervention, and 98% (227/231) of ART clients in the index HIVST arm were comfortable demonstrating HIVST to their index partner.

**Table 3:** Acceptability of the intervention and adverse events, reported by ART clients (*n* = 364).

Variable	PRS arm	HIVST arm	RR (95% CI)	*p*-Value	aRR (95% CI)^^^	*p*-Value
*n* (%)	*n* (%)
**Acceptability among ART clients**						
**Among those who delivered intervention (*n* = 329)**						
Comfortable explaining the intervention	97/98 (98.9%)	228/231 (98.7%)	0.99 (0.99, 1.01)	0.528	0.99 (0.98, 1.01)	0.653
Female ART client	72/73 (98.6%)	177/179 (98.9%)	1.00 (0.98, 1.03)	0.789	1.01 (0.97, 1.05)	0.702
Male ART client	25/25 (100%)	51/52 (98.1%)	-	-	-	-
**Index HIVST arm only**						
Comfortable demonstrating how to use HIVST	-	227/231 (98.3%)	-	-	-	-
Female ART client	-	176/179 (98.3%)	-	-	-	-
Male ART client	-	51/52 (98.1%)	-	-	-	-
**Adverse events** *						
Experienced psychological IPV in the past 4 weeks	9/107 (8.4%)	21/257 (8.2%)	0.97 (0.56, 1.64)	0.914	1.00 (0.56, 1.78)	0.998
Female ART client	9/81 (11.1%)	18/203 (8.9%)	0.80 (0.48, 1.34)	0.393	0.84 (0.45, 1.58)	0.588
Male ART client	0/26 (0%)	3/54 (5.6%)	-	-	-	-
Experienced psychological IPV due to the intervention	4/107 (3.7%)	3/257 (1.2%)	0.31 (0.06, 1.67)	0.173	0.30 (0.05, 1.79)	0.187
Female ART client	4/81 (4.9%)	3/203 (1.5%)	0.30 (0.06, 1.57)	0.153	0.30 (0.05, 1.68)	0.169
Male ART client	0/26 (0%)	0//54 (0%)	-	-	-	-
Termination of relationship (divorce or end of relationship)	7/107 (6.5%)	19/257 (7.4%)	1.13 (0.91, 1.41)	0.273	1.19 (1.01, 1.40)	0.043
Female ART client	5/81 (6.2%)	17/203 (8.4%)	1.36 (0.74, 2.49)	0.325	1.37 (0.79, 2.38)	0.267
Male ART client	2/26 (7.7%)	2/54 (3.7%)	0.48 (0.10, 2.28)	0.357	0.46 (0.08, 2.84)	0.406

*Individuals who had already tested HIV+ and decided to test again.

^^^Adjusted for age and marital status.

aRR, adjusted risk ratio; ART, antiretroviral therapy; CI, confidence interval; HIVST, HIV self-test; IPV, interpersonal violence; PRS, partner referral slip; RR, risk ratio.

Adverse events experienced by ART clients did not vary by arm. Eight percent (30/364, *p* = 0.940) of ART clients across both arms reported psychological IPV in the 4 weeks after study enrollment. Four percent (4/107) of those in the PRS arm and 1% (3/257) in the HIVST arm reported psychological IPV events that they believed were due to the assigned intervention arm (*p* = 0.116). All ART clients who reported IPV were female. There were no reports of physical or sexual IPV. Termination of relationship status was 7% (26/364) in both arms (*p* = 0.774).

### Feasibility and acceptability of index HIVST by partners

We conducted a survey with a subset of partners in the index HIVST arm who attempted to use HIVST to understand HIVST user acceptability and feasibility (*n* = 161). Mean age of surveyed partners was 41 years (SD: 11.6) and 73% (117/161) were male (Table 4). Among partners who tried to use the HIVST (*n* = 126), 16% (20/126) reported challenges understanding HIVST instructions and 10% (13/126) were unable to interpret HIVST results. Among those able to interpret their test results (*n* = 116), only 3% (3/116) did not trust test results (and all reported HIV–negative HIVST results).

**Table 4:** Index partner recruitment and characteristics of partners in the index HIV self-test arm and completed a follow-up survey (*n* = 257).

Variable	HIVST partners
	*n* (%)
**Recruitment and survey response rate**	
Total recruited via passive study invitation cards	257 (100%)
Partners who completed a follow-up survey	161 (63%)
**Demographic characteristics of those who completed a survey (*n* = 161)**	
Male	117 (72.7%)
Mean age, years (SD)	41 (11.6)
**HIVST testing**	
Received HIVST	148 (91.9%)
Used HIVST	126/148 (85.1%)
**Acceptability among index partners in HIVST arm (*n* = 126)**	
Received help from ART client to use HIVST	79 (62.7%)
Unable to interpret HIVST test results	10 (7.9%)
Partner had difficulty with the following:	
Understanding HIVST instructions	20 (15.9%)
Interpreting kit result	13 (10.3%)
Keeping the test result private	1 (0.8%)
**Among those who interpreted HIVST kit (*n* = 116)**	
Did NOT trust accuracy of test results	3 (2.6%)

ART, antiretroviral therapy; HIVST, HIV self-test; SD, standard deviation.

### Cost analysis

The cost per test provided was lower for PRS ($0·84) than for HIVST ($2·34) (Table 5). This is explained by the fact that not everyone who received a PRS received a test, whereas every HIVST distributed incurs the cost of an HIVST kit. The cost per person newly aware of their positive status was lower for HIVST than for PRS ($16·06 and $19·35, respectively) given that more people used the self-test kit than followed up using their partner referral slip (PRS). However, the cost per new ART initiate was far greater for HIVST as compared to PRS ($84·15 versus $24·46). This is driven by higher cost of HIVST kits and individual partners who tested positive using HIVST but did not follow-up at a health care facility for a confirmatory test and ART initiation.

**Table 5:** HIV testing uptake among index partners, reported by ART clients (*n* = 364).

Costing	PRS arm	HIVST arm
**Costing inputs**		
Cost of counseling for PRS or index HIVST distribution*	$0·15	$0·23
Cost per HIVST kit	-	$2·00
Cost of testing negative at health care facility**	$2·29	$2·29
Cost of testing positive at health care facility**	$3·85	$3·85
**Costing outcomes**		
Cost per test provided	$0·84	$2·34
Cost per person aware of HIV status	$19·35	$16·06
Cost per ART initiate	$24·46	$84·15

*All-inclusive costs (human resources, training, equipment, overhead).

**All-inclusive costs (human resources, training, equipment, test kits, overhead).

ART, antiretroviral therapy; HIVST, HIV self-test; PRS, partner referral slip.

## Discussion

In this RCT, we found that index HIVST among the primary partners of ART clients dramatically increased the proportion of partners tested for HIV. The index HIVST arm showed a 167% increase in testing uptake as compared to standard PRSs, achieving testing coverage of 71% among sexual partners. Due to increased testing coverage, index HIVST resulted in a 211% increase in the number of partners identified as living with HIV as compared to PRS. After 12 months, 14/30 (47%) of those newly diagnosed in the HIVST arm and 3/4 (75%) of those in the PRS arm initiated ART. However, the HIVST arm also showed nearly double the proportion of total partners initiating ART—even with lower initiation rates—due to dramatic increases in testing coverage (5·4% of the total HIVST sample initiating ART versus 2·8% of the total PRS sample). Findings suggested that index HIVST can effectively increase testing uptake and ART coverage among partners of ART clients, although additional work is needed to improve linkage to ART after HIVST use. Routine testing for individuals at higher risk of HIV acquisition is important for both treatment and prevention efforts. Utilizing index HIVST approaches may be an important step to facilitating routine testing as part of individuals self-care practices [35].

The intervention was cost-efficient for new diagnoses, and the cost per newly diagnosed individual was low compared to other testing modalities that target general populations (such as community screening, facility-based HIVST, among others) [36]. This is likely due to the high probability of index partners being positive as compared to people tested through other modalities. The main cost drivers were the cost of the self-test itself, as compared with the cost of a patient referral slip. However, we found that only 9% of ART clients screened had partners who were eligible for index HIVST. This suggests that while index HIVST is effective in the Malawi setting, the intervention’s reach at a national level may be narrow and limit the overall impact of index HIVST on national testing outcomes.

A sub-analysis of our data showed large testing differences by sex—in the HIVST arm 89% of female partners tested versus 67% of male partners. This disparity is important as index testing represents a key strategy for reaching men, who comprised over 78% of eligible index partners and have few additional entry points into HIV services [37–39]. Strategies to engage men must be prioritized, including HIV testing and linkage to additional services after testing [11]. A package of strategies may be needed in order to optimally reach male partners, such as index HIVST plus peer support for men or male targeted promotional messaging [29,38]. In addition, other entry points such as outpatient departments, the only facility-based entry point reached by most men, may need to be utilized further to reach men.

Index HIVST was highly acceptable among ART clients, with no differences in adverse events reported by arm. Our findings are similar to other secondary HIVST distribution strategies with both HIV–negative and status unknown women in Kenya and Malawi [21–23] and the few interventions with women living with HIV [25,26]. High acceptability may have been due to the fact that ART clients in our trial were on ART for a mean of 5 years and over 90% had already disclosed to their partner prior to study enrollment. Most relationships were highly stable—the majority were married, had children together, and were in a relationship for a mean of 9 years—which may result in greater trust and confidence in their relationship, facilitating safe HIVST distribution. Other studies in Uganda and South Africa show that women who have not yet disclosed their HIV–positive status may be apprehensive about HIVST distribution and fear negative repercussions from male partners [26,40]. Additional research is needed to assess how index HIVST performs among individuals in non-married and/or unstable relationships, particularly when the ART client has not yet disclosed their status.

Eight percent of index partners who used HIVST were unable to interpret their test result. Understanding the usability of HIVST within index testing is critical to ensuring the strategy is effective and scalable in low-resource settings, particularly where health literacy is low. The limited data available on the usability of secondary HIVST show that most partners are able to complete an HIVST test successfully, but some require guidance, desire pre-test counseling, or mistrust HIVST results [24,38]. Additional research is needed to identify strategies that facilitate accurate test interpretation within secondary distribution models, such as additional visual aids and/or wider community sensitization about HIVST.

We examined ART initiation among HIVST users longitudinally. Twelve months after enrollment, we found that only 47% of index partners who had a reactive HIVST kit initiated ART, and the vast majority of these individuals did so within the first 3 months after testing. Our experience is similar to other published data on secondary HIVST distribution, where linkage and initiation rates range from 23% to 68% [27–30]. There are several potential explanations for low initiation rates within HIVST strategies. First, HIVST users may already be on ART but retest because HIVST is convenient and does not require further conversations with providers, therefore removing the need for ART initiation. Although we are unable to confirm this hypothesis, other research in the region shows those with a known HIV status do retest for numerous reasons [41]. Second, among those who did not previously know their status (or were aware but not on ART), simply knowing one’s status may not be enough to overcome traditional barriers to HIV service utilization (such as stigma, time and money required for facility visits, fear of unwanted disclosure at facilities, and fear of rude providers) [42,43]. Qualitative data show that male HIVST users desire additional knowledge, opt-out options for linkage to care, and positive examples and support from men who have successfully initiated HIV treatment [43]. Linkage to prevention services should also be prioritized among those testing HIV–negative; however, it is unclear what interventions can support linkage to prevention efforts, especially for men in the region [44]. For both treatment and prevention, combined interventions are likely needed that couple index HIVST with strategies that facilitate linkage in order to address these ongoing barriers to care. Potential linkage interventions to optimize the impact of index HIVST include outside facility services, private and fast services, strong Welcome Back strategies (for those living with HIV), and peer counseling [43,45,46].

This study has several limitations. First, ART clients who did not complete a 4-week endline survey were excluded from the analyses because it was impossible to determine primary and secondary outcomes, which may result in bias if those retained were more likely to have used and be satisfied with the intervention. Second, we rely on secondary reports from ART clients to determine primary outcomes for their sexual partners. Secondary reports are likely to underestimate the impact of index HIVST since partners may use an HIVST kit, or receive additional HIV services, without the ART client’s knowledge. Third, our primary outcome (HIV testing) was measured 4 weeks after enrollment of the ART client, allowing only a short period for outcome attainment. Fourth, we did not collect data on linkage to prevention services for those who tested HIV–negative. It is critical that future HIV testing interventions take a status neutral approach [35], and future index HIVST research should examine how to promote both prevention and treatment services after use of HIVST. Despite this limitation, we found high rates of testing in the index HIVST arm and similar rates of testing in the PRS arm as compared to other studies. While the cost of provision through this trial may have different slightly to the cost of HIV testing in the HIVST trial from where the costing data were obtained, the magnitude of difference in the cost of the strategies is unlikely to differ. Finally, our sample size is small and the vast majority of ART clients were married and had already disclosed their HIV status to their partner. Additional research is needed to assess if findings are replicable in other facility types and other regions. Due to the small sample size, we had limited power to detect statistical differences in secondary outcomes.

Index HIVST greatly increased HIV testing among sexual partners of ART clients without increasing adverse events. The intervention was cost-efficient for new diagnoses, but strategies to improve ART initiation after receiving reactive HIVST results are needed. Further research is needed to understand the role of index HIVST among non-stable partners and what strategies can optimize testing among male partners and ART initiation more broadly.

## Supporting information

S1 CONSORT Checklist Consolidated Standards of Reporting Trials (CONSORT) Checklist. (DOC)

S1 Text Study protocol. (DOCX)

S1 Table Sensitivity analyses using true intention-to-treat. (DOCX)

S2 Table Sensitivity analyses with additional covariates, HIV testing uptake among partners. (XLSX)

S3 Table Baseline characteristics among those with a primary outcome, reported by ART client. (DOCX)

S1 Fig Kaplan–Meier curve showing time to antiretroviral therapy initiation among HIV self-test users who test positive for HIV. (DOCX)

## Abbreviations

aRR: adjusted risk ratio
ART: antiretroviral therapy
CI: confidence interval
HCW: health care worker
HIVST: HIV self-test
IPV: interpersonal violence
LTFU: lost to follow-up
PRS: partner referral slip
RCT: randomized controlled trial
RR: risk ratio
SD: standard deviation
