# Supplementary material for: Effect of index HIV self-testing for sexual partners of clients enrolled in antiretroviral therapy (ART) programs in Malawi: A randomized controlled trial
Source: PLoS Med. 2023 Aug 4;20(8):e1004270. doi: 10.1371/journal.pmed.1004270 (PMC10403056; doi:10.1371/journal.pmed.1004270)
Supplement: S1 Text — (DOCX) [file pmed.1004270.s002.docx]

****Use of HIV Self-Test Kits to Increase Identification of Index Partners in Malawi****

Sponsored by:

**USAID/PEPFAR**

**Protocol Chair: Kathryn Dovel, PhD**

**Protocol Vice Chairs: Risa Hoffman MD, MPH and Mike Nyirenda**

Version 1.2

9 January 2016

****Use of HIV Self-Test Kits to Increase Identification of HIV-Infected Individuals and Their Partners****

TABLE OF CONTENTS

[Abbreviations and Acronyms 3](#_Toc130807538)

[1 Introduction 5](#_Toc130807540)

[1.1 Background 5](#_Toc130807541)

[1.2 Prior Research on Self-Testing 5](#_Toc130807542)

[1.3 Rationale 5](#_Toc130807543)

[1.4 Hypotheses 5](#_Toc130807544)

[1.5 Objectives 6](#_Toc130807545)

[1.6 Study Population 6](#_Toc130807546)

[1.7 Study Design 6](#_Toc130807547)

[1.8 Study Procedures 8](#_Toc130807548)

[1.9 Statistical Methods 10](#_Toc130807549)

[1.10 Cost and Cost-Effectiveness Methods 11](#_Toc130807550)

[2 Data Handling and Record Keeping 12](#_Toc130807551)

[2.1 Data Management Responsibilities 12](#_Toc130807552)

[3 Site Monitoring 12](#_Toc130807553)

[4 Safety Assessment, Monitoring, and Reporting 12](#_Toc130807554)

[5 Human Subjects Protections 13](#_Toc130807555)

[5.1 Institutional Review Board/Ethics Committee Review and Approval Including Informed Consent 13](#_Toc130807556)

[5.2 Potential Benefits 13](#_Toc130807557)

[5.3 Potential Risks and Discomforts 13](#_Toc130807558)

[5.4 Reimbursement/Compensation 13](#_Toc130807559)

[5.5 Privacy and Confidentiality 13](#_Toc130807560)

[5.6 Management of New Information Pertinent to Study Participation 14](#_Toc130807561)

[6 Administrative Procedures 14](#_Toc130807562)

[6.1 Regulatory Oversight 14](#_Toc130807563)

[6.2 Study Implementation 14](#_Toc130807564)

[6.3 Protocol Deviation Reporting 14](#_Toc130807565)

[7 Publications 14](#_Toc130807566)

[8 References 15](#_Toc130807567)

# Abbreviations and Acronyms

ARV: antiretrovirals

ART: antiretroviral therapy

CMC: clinical management committee

DSMB: data safety and monitoring board

CRF: case report form

EC: ethics committee

HDA: HIV diagnostic assistant

HSA: health surveillance assistant

IRB: Institutional Review Board

NHSRC: National Health Science Research Committee

PEPFAR: President’s Emergency Plan for AIDS Relief

PID: Patient Identification Number

PIH: Partners in Hope

SOC: standard of care

SOP: Standard Operating Procedure

STI: sexually transmitted infection

TA: Technical Assistance

USAID: United States Agency for International Development

# Introduction

## Background

In order to reach the UN 90-90-90 goals, innovative solutions must be approached to identify and test individuals living with HIV. UNAIDS and the CDC have identified employing Partner Notification Services, also known as Contact Tracing, to augment HIV case finding [[1](#_ENREF_1), [2](#_ENREF_2)]. The feasibility and effectiveness of Partner Notification Services in Sub-Saharan African countries has been demonstrated with much success [[3-7](#_ENREF_3)]. Within Malawi and Cameroon, active partner notification was effective with high rates of partner testing, 51% and 66.8% respectively, with positivity rates between 50-64% [[3](#_ENREF_3), [4](#_ENREF_4)]. Partner notification services have the potential for high impact in new case identification as the rate of HIV cases among partners has been shown to be tenfold higher than the national prevalence [[2-4](#_ENREF_2)].

Early case identification is also key in preventing late stage patient presentation with high morbidity and less efficacious treatment outcomes. The case finding effectiveness of Partner Notification Services can result in earlier diagnosis of individuals, earlier initiation of ART, and prevention of HIV transmission. However, in the context of Partner Notification Services, percent return rates of men during passive referral is half that seen by women partners [[3](#_ENREF_3)]. Strategies to improve testing of men are needed [[8-10](#_ENREF_8)].

## Prior Research on Self-Testing

A recently introduced method of HIV self-test screening within Malawi has shown high testing uptake among men. A study piloting the use of HIV self-testing demonstrated high uptake in adolescent men (89.3% in men aged 16-19 years of age) with rates remaining as high as 60% up to age 39 (compared to only 42% of the general male population who have been tested in the previous year) [[11](#_ENREF_11)]. Self-test screening has also shown the benefit of promoting partner testing and facilitating open discussion for disclosure [[12](#_ENREF_12),[13](#_ENREF_13)].

Overall, feasibility and uptake of self-test screening has been successfully demonstrated within Malawi allowing self-test screening to serve as a new key tool in increasing access to testing [[11](#_ENREF_11),[12](#_ENREF_12),[14-16](#_ENREF_14)]. HIV self-test screening has been shown to be accurate, safe, and cost/quality of life analysis has also demonstrated its usefulness [[11](#_ENREF_11),[12](#_ENREF_12),[14](#_ENREF_14),[16-19](#_ENREF_16)]. Self-test kits have not been studied for partner testing.

## Rationale

While self-testing and partner notification strategies have been studied separately, these strategies have never been examined jointly as a means to improve partner disclosure and testing. We propose to study the role of self-testing in identifying HIV-positive individuals and to determine whether self-test kits can be given to index clients as a means to facilitate partner disclosure and partner testing.

## Hypotheses

- Self-test kits given to index clients will (1) increase the number of index partners who test for HIV; (2) increase yield of partner testing, and (3) be cost-effective compared to standard of care for partner notification and testing, and will (4) increase partner disclosure by the index client.

## Objectives

### Primary Objectives:

- To determine whether providing index clients with HIV self-test kits for partners results in a greater number of partners tested as compared to standard of care for partner notification and testing (referral slip)
- To determine whether providing index partners with HIV self-test kits is cost-effective compared to standard of care using referral slips

### Secondary Objectives:

- To assess the acceptability of giving self-test kits to index clients for partner testing
- To determine whether providing index partners with HIV self-test kits is superior standard of care in regard to identifying HIV+ partners (yield of testing)
- To determine whether providing index partners with HIV self-test kits is superior to standard of care in regard to linkage rates among those who identify as HIV-positive
- To determine whether providing self-test kits to index clients and their partner is superior to standard of care in regard to partner disclosure by the index client

## Study Population

This study will be conducted among approximately 360 individuals 15 years or older who have at least one sexual partner with unknown HIV status.

### Inclusion Criteria

All of the criteria listed below must be met in order for an individual to be included in this study.

- - At least 15 years of age or older
  - Willing and able to provide informed consent for participation in this study
  - HIV-positive
  - Have at least one sex partner in the catchment area with an unknown HIV status at the time of study enrollment (defined as never testing for HIV or testing HIV negative more than 6 months ago)
  - No current or prior history of intimate partner violence
  - No fear of intimate partner violence as a consequence of participating in the study

### Exclusion Criteria

Individuals will be excluded from the study if any of the following are identified during screening or any other time during the study:

- - HIV-negative
  - Younger than 15 years of age
  - No sex partners with unknown HIV status in the catchment area at the time of study enrollment
  - Experienced intimate partner violence with current sexual partner
  - Fear of intimate partner violence as a consequence of participating in the study
  - Unwilling or unable to provide informed consent

## Study Design

Individually randomized, unblinded control trial.

### Description of Randomization and Study Arms

We will randomize eligible ART clients 1:2.5 to standard of care or partner self-testing arms.

#### Description of Study Arms

1. Standard of care arm: Facilities randomized to the standard of care arm will use MOH approved referral mechanisms, without any intervention from the study team. Standard of care quality will vary by facility and may include some level of partner referral services for index partners such as referral slips. Referral slips may be given at HIV testing or ART clinics. Sites will be asked to counsel clients to have their partners bring referral slips back to the clinic to document the number of partners presenting for HIV testing. A random subset of HIV-positive clients in the standard of care arm will complete an anonymous survey to answer questions about whether they used referral slips and whether they know if partners came to a health facility for testing.
2. Partner self-testing arm: Facilities randomized to partner self-testing will have study staff give self-test kits to index clients to distribute to every sexual partner with unknown status who lives in the facility catchment area within the prior 12 months. They will also be given self-test kits for themselves so they can participate in couples testing if they choose. All index clients will be counseled on the importance of partner notification and testing and receive a demonstration on how to perform the self-test, so they can show partners. Self-test kits will contain information on how to use the test and a referral card. The referral card will contain information on where to present for confirmatory testing and where partners can receive ART, including a map to the facility. Index clients will be counseled to tell their partner to drop the completed test in a lockbox at a house in the local community (home of the health surveillance assistant) or in a lockbox at the local health facility. Written instructions, picture instructions, and maps will also be provided to the index client to give to the index partner. Index clients will also be counseled to encourage partners to present to the facility for routine HDA testing if they are unable or uncomfortable completing the self-test.

### Recruitment, Screening, and Enrollment Process

Individuals will be recruited and initially screened by providers (HDAs and/or ART providers) who will identify HIV-positive clients 15 years of age and older with at least one partner who has unknown HIV status. Screening will be performed in a private space where no other clients or staff can hear the exchange of information. Eligible index clients will be referred to study staff for further screening (see 2.2.1 and 2.2.2 for inclusion/exclusion) and those who provide written informed consent will be enrolled and randomized.

### Informed Consent

Written informed consent will be obtained from all ART clients before any study-specific procedures are performed. The informed consent process will include information exchange, detailed discussion, and assessment of understanding of all required elements of informed consent, including the potential risks, benefits, and alternatives to study participation. The process will emphasize the randomized nature of the study and the differences that participants may experience as part of the study relative to current local standards of care.

### Participant Withdrawal or Termination from the Study

Individuals may withdraw from the study at any time. Participants may also be terminated from the study by the site investigator or designee under the following circumstances:

- Site investigator or designee determines that continued participation in the study would be unsafe or otherwise not in the best interest of the participant
- The study is stopped or canceled by the sponsors, government or regulatory authorities, or site IRBs

## Study Procedures

### Standard of Care Arm

#### 3.4.4.1 Baseline Visit for Index Client

After written informed consent has been obtained, study staff will collect basic socio-demographics (including age, gender, marital status, and number of partners), HIV characteristics (date of HIV diagnosis, whether on ART and date started), and sexual partner history.

*3.4.1.1 Provision of Services*

No intervention activities will be conducted. Standard of care procedures involve counseling HIV-positive individuals about the importance of partner testing and providing ‘referral slips’ to the index client to distribute to sex partners. Partner testing will follow Ministry of Health National Guidelines using serial testing with Determine and Uni-Gold. Those that test positive will be counseled and referred to ART services following routine care.

*3.4.1.2* *Data Collection*

A random sample of individuals will be screened and consented as they exit their ART visit. Individuals who consent will be surveyed to determine whether they were offered a referral slip at any of their recent ART visits (within the most recent two ART visit or ~last six months). If they did receive a referral slip for their partner, follow-up questions will include whether they used the slip, whether their partner tested, whether they know the result of their partner’s test, and for partners who test HIV positive, whether they know if their partner linked to care. HIV testing registers will also be reviewed to measure the number of individuals tested through partner referrals and the proportion of partner referral tests who were identified as HIV-positive.

All registry data will be anonymized. Records from partner notification clients aged 15 years and older will be included in the study. For those who are HIV-positive, the HTC and ART registers will be reviewed to determine if the patient started ART and presented for their four-week follow-up visit.

#### 3.4.4.4 Follow-up Visit of Index Clients

All index clients will be seen by study staff when they come for their next ART follow-up visit. A brief survey will be performed to collect information about whether the client disclosed their HIV status to their sex partner(s), whether they gave the HIV test referral slip to their partner(s), and knowledge about whether partners completed an HIV test, knowledge of their partner’s test result, and knowledge about their partner’s linkage to additional HIV services. Index clients who do not return for their ART follow-up visit (more than two weeks late) will be traced through standard site protocols and surveyed when/if they return to care.

### Partner Self-Testing Arm

#### 3.4.4.1 Baseline Visit for Index Client

After written informed consent has been obtained, study staff will collect basic socio-demographics (including age, gender, marital status, and number of partners), HIV characteristics (date of HIV diagnosis, whether on ART and date started), and sexual partner history.

The study staff will counsel the index client on how to administer an HIV self-test and will provide one self-test kit for each sexual partner within the last 12 months with unknown status (or HIV-negative more than 6 months ago) who lives within the catchment area of the facility and for whom the index client is willing to provide the test kit (up to three sexual partners). Index clients will also be given self-test kits for themselves if they wish to use them to complete couples counseling. All HIV self-test kits will be labeled with a unique ID linked to the index client participant. The ID will also be used to link index client survey responses with returned self-test kits.

The number of partners and the number of kits provided will be documented by study staff. Each self-test kit will be in a package that also contains a referral card. The referral card can be used for (1) those who test HIV-positive and need to present for confirmatory testing and link to care, (2) those who do not want to use the self-test kit and would rather test with an HDA, or (3) clients who experience difficulty or discomfort with the self-test kit and choose to test at a facility. The referral card will include information about the facility, including a map. The card will also contain a notation that if it is returned to another facility, the facility should contact the study team so that the card can be collected and recorded as “returned”. Study staff will periodically visit nearby testing sites to determine if any referral cards have been returned to those sites.

*3.4.4.2 Partner Self-Testing*

Index clients will provide their partners with the following instructions verbally. The following instructions will also be provided on the referral form in picture form and in the local language:

1. Complete HIV self-test
2. If the self-test is positive, bring the self-test kit and referral card to the clinic for confirmatory testing and linkage to care.
3. If the self-test is positive and the client does not wish to present for further care, the self-test kit should be disposed of in the lockbox at the HSA house.
4. If the test is negative, the client will be instructed to dispose the self-test kit in a lockbox at the HSA house in the local community or may bring the kit to the lockbox at OPD/STI.

Any individual who has difficulty with the self-test kit or needs help with interpretation can come to the facility with the kit and the referral card and be seen by an HDA for assistance and/or standard of care HIV testing.

#### 3.4.4.4 Follow-up Visit of Index Clients

All index clients will be seen by study staff when they come for their next ART follow-up visit. A brief survey will be performed to collect information about whether the client disclosed their HIV status to their sex partner(s), whether they gave self-test kits to their partner(s), and knowledge about whether partners completed the test, knowledge of their partner’s test result, and knowledge about their partner’s linkage to care. We will also survey the index client to determine whether they were able to successfully show their partner(s) how to use the kit and whether any gender-based violence or fear of gender-based violence resulted from use of the self-tests. Index clients who do not return for their ART follow-up visit (more than two weeks late) will be traced through standard site protocols and surveyed when/if they return to care.

A random sample of 30 index clients (15 men and 15 women) will be selected and asked to participate in semi-structured, in-depth interviews to explore the feasibility and acceptability of providing self-test kits to partners, including challenges with disclosure and questions around harm or perceived risk of harm due to distributing the kits.

## Statistical Methods

### Outcome Measures

#### 3.5.1.1 Sample Size and Statistical Analysis for Primary Outcome Measures

Assuming a type I error of 0.05, and sample sizes of 110 in standard of care and 250 in HIVST arms (randomized 1:2.5), we have >90% power to detect a difference in partner testing coverage of 40% in standard of care and 60% in HIVST arms. Because we are uncertain about the proportion of partners who will test under each arm, we have considered a range of possibilities, with the majority of scenarios providing at least 87% power to detect a difference between the two arms (standard of care and self-testing).

*3.5.1.1 Analysis Plan*

We will calculate descriptive statistics, including mean/median, variation (standard deviation, kurtosis), range, and frequency distributions for the demographic and clinical characteristics, overall and by study arm. The outcomes of interest include the proportion of partners tested and HIV-positivity of partners. Differences in the prevalence of each of the outcomes of interest will be examined by study arm as well as by gender. The differences will be evaluated using t-tests, Mann-Whitney U test (or other non-parametric tests), chi-square methods, and Fisher’s exact test as appropriate. Separate models will be developed for each outcome of interest and covariates of interest include patient characteristics (e.g., age, gender) and other structural and contextual factors.

#### Qualitative Analysis for Secondary Outcome Measures

Our secondary outcomes measured will be feasibility and acceptability of partner self-testing, privacy concerns, problems with performing or interpreting self-tests, and perceived harms or benefits resulting from self-testing. Qualitative methods (semi-structured in-depth interviews) will be conducted on a subset of index clients (n=30) from the partner self-testing arm.

## Cost and Cost-Effectiveness Methods

It is expected that partner self-testing will be cost-effective compared to standard of care. For facilities, fewer HDA visits by patients should save the time of HDA staff. Partner self-testing may also lead to an increase in HIV-positive individuals identified because clients may be more comfortable doing the test in a private place. The study will estimate differences in overall costs. The average cost per successful primary outcome (index partner completes an HIV test) and secondary outcome (index partner initiate ART) will be calculated.

Using the average cost per patient as described above, we will then estimate the cost for each newly identified HIV-positive partner. We will compare average cost per HIV-positive individual identified between study arms. To provide information for HIV program budgets, we will also estimate the annual cost of providing HIV testing under the two main strategies being evaluated (standard of care and self-testing), independent of outcomes.

### Costs to Provider

Costs will be measured from the provider perspective. We will use micro-costing methods to estimate the cost of HIV testing in all three study arms [[20-22](#_ENREF_20)]. We will first create an inventory of all the resources used to achieve the observed study outcomes. Resources to be captured will include:

- HIV self-test kits
- Standard of care HIV testing supplies
- Other services provided (e.g. counseling interactions)
- Fixed costs of patient care (building space, equipment, human resources)

For each study patient, the quantity (number of units) of resources used will be determined. Unit costs of resources, which are not human subjects data, will be obtained from external suppliers and the site’s finance and procurement records and multiplied by the resource usage data to provide an average cost per study patient across centers in each study arm. Costs will be reported as means (standard deviations) and medians (IQRs) in USD, using the exchange rate prevailing during the follow up period.

### Cost-Effectiveness

Using the average cost per patient as described above, we will then estimate the cost per outcome achieved in each arm. The main measure of effectiveness for the cost-effectiveness analysis will be both the primary study outcome of proportion of partners tested as well as yield (newly identified HIV-positive partners of index cases). We will calculate the difference in cost divided by the difference in effectiveness among study arms for both effectiveness measures.

The price of the self-test itself is currently uncertain because it is not widely available in Malawi. As such, we will conduct a sensitivity analysis where all costs and outcomes remain constant, and the price of self-tests alone is varied. This analysis will then provide a plausible range of the cost-effectiveness of partner self-testing.

### Monitoring by the Protocol Team

The Protocol Team is responsible for continuous monitoring of study progress, including timely achievement of key milestones and quality of study conduct.

The team will closely monitor participant accrual based on reports that will be generated at least monthly. For any site that falls short of its accrual projections, the team will communicate with the site research assistants and leadership to identify the barriers the site has encountered and the operational strategies and action plans to address these.

The Protocol Team will similarly review key indicators of the quality of study conduct (e.g. data quality and data completeness) based on reports and take action with study sites as needed to ensure high quality study conduct.

# Data Handling and Record Keeping

## Data Management Responsibilities

Study sites must maintain adequate and accurate research records containing all information pertinent to the study for all screened and enrolled participants, including CRFs and supporting source data. Depending on capacity and infrastructure of sites/regions, data will either be collected by hand and entered into a database or collected electronically with data uploaded to a database.

All data must be transferred to the central database within timeframes specified in the forms’ instructions; queries must also be resolved in a timely manner.

# Site Monitoring

Site monitors will visit study sites to inspect study facilities and review participant study records including consent forms and CRFs, to ensure protection of study participants, compliance with the IRB/EC approved protocol, and accuracy and completeness of records. Site investigators will make study facilities and documents available for inspection by the monitors.

# Safety Assessment, Monitoring, and Reporting

Participant safety will be carefully assessed, monitored, and reported at multiple levels throughout this study.

# Human Subjects Protections

## Institutional Review Board/Ethics Committee Review and Approval Including Informed Consent

Prior to study initiation, site investigators must obtain IRB/EC review and approval of this protocol and ICFs; subsequent to initial review and approval, IRBs/ECs must review the study at least annually.

All IRB/EC policies and procedures must be followed, and complete documentation of all correspondence to and from the IRBs/ECs must be maintained in site essential document files. Sites must submit documentation of both initial review and approval and continuing review to the EQUIP Protocol Team.

Informed consent will be obtained before any study-specific procedures are performed. The informed consent process will include information exchange, detailed discussion, and assessment of understanding of all required elements of informed consent, including the potential risks, benefits, and alternatives to study participation. The process will emphasize the randomized nature of the study and the differences that participants may experience as part of the study relative to current local standards of care.

## Potential Benefits

There may be no direct benefit to participants who take part in this study, though participants who are randomly assigned to the self-testing study arm may benefit from the convenience and privacy of completing an HIV test at a location and time of their choosing. Information learned in this study may be of benefit to participants and others in the future, particularly information that may lead to optimized testing guidelines.

## Potential Risks and Discomforts

Most study procedures are routine clinical care associated with minimal to no risk in participants. Participants will be screened for risk of intimate partner violence, and those with any risk will be excluded.

## Reimbursement/Compensation

Participants who complete follow-up surveys and qualitative interviews will be reimbursed/compensated for their time and any transport costs. The amount of reimbursement will be deemed appropriate by the Malawi NHSRC.

## Privacy and Confidentiality

All study procedures will be conducted in private, and every effort will be made to protect participant privacy and confidentiality to the extent possible. Participant information will not be released without written permission to do so except as necessary for review, monitoring, and/or auditing.

All study-related information will be stored securely. Participant research records will be stored in locked areas with access limited to study staff. All laboratory specimens, CRFs, and other documents that may be transmitted off-site will be identified by PID only. Likewise, communications between study staff and protocol team members regarding individual participants will identify participants by PID only.

Study sites are encouraged to store study records that bear participant names or other personal identifiers separately from records identified by PID. All local databases must be secured with password-protected access systems. Lists, logbooks, appointment books, and any other documents that link PID numbers to personal identifying information will be stored in a separate, locked location in an area with limited access.

## Management of New Information Pertinent to Study Participation

Study staff will provide participants with any new information learned over the course of the study that may affect their willingness to participate.

# Administrative Procedures

## Regulatory Oversight

This study is sponsored by USAID/PEPFAR and implemented through Partner in Hope (PIH)-EQUIP. PIH-EQUIP staff will perform monitoring visits. As part of these visits, monitors will inspect study-related documentation to ensure compliance with all applicable regulatory requirements.

Site-specific ICFs will be reviewed and approved by the EQUIP key personnel, and sites will receive an Initial Registration Notification from EQUIP that indicates successful completion of the protocol registration process. A copy of the Initial Registration Notification should be retained in the site's regulatory files.

For any future protocol amendments, upon receiving final IRB/EC and any other applicable regulatory entity approvals, sites should implement the amendment immediately. Sites are required to submit an amendment registration packet to the EQUIP Protocol Team. EQUIP key personnel will review the submitted protocol registration packet to ensure that all the required documents have been received.

## Study Implementation

Study implementation at each site will also be guided site-specific standard operating procedures (SOPs). These SOPs should be updated and/or supplemented as needed to describe roles, responsibilities, and procedures for this study.

## Protocol Deviation Reporting

All protocol deviations must be documented in participant research records. Reasons for the deviations and corrective and preventive actions taken in response to the deviations should also be documented.

Deviations should be reported to site IRBs/ECs and other applicable review bodies in accordance with the policies and procedures of these review bodies. Serious deviations that are associated with increased risk to one or more study participants and/or significant impacts on the integrity of study data must also be reported to the Protocol Team as soon as possible.

# Publications

All presentations and publications of data collected in this study are governed by EQUIP and USAID/PEPFAR policies.

# References

1. Bocour A, Renaud TC, Udeagu C-CN, Shepard CW. HIV partner services are associated with timely linkage to HIV medical care. AIDS. 2013;27(18):2961-3. doi: 10.1097/qad.0000000000000031. PubMed PMID: 00002030-201311280-00016

2. UNAIDS: Global HIV/AIDS response: epidemic update and health sector progress towards universal access: progress report 2011. Accessed 1 March 2014.

3. Brown LB, Miller WC, Kamanga G, Nyirenda N, Mmodzi P, Pettifor A, et al. HIV partner notification is effective and feasible in sub-Saharan Africa: opportunities for HIV treatment and prevention. J Acquir Immune Defic Syndr. 2011;56(5):437-42. Epub 2011/11/03. doi: 10.1097/qai.0b013e318202bf7d. PubMed PMID: 22046601; PubMed Central PMCID: PMCPMC3207356.

4. Henley C, Forgwei G, Welty T, Golden M, Adimora A, Shields R, et al. Scale-up and case-finding effectiveness of an HIV partner services program in Cameroon: an innovative HIV prevention intervention for developing countries. Sex Transm Dis. 2013;40(12):909-14. Epub 2013/11/14. doi: 10.1097/olq.0000000000000032. PubMed PMID: 24220349; PubMed Central PMCID: PMCPMC4521599.

5. Hogben M, McNally T, McPheeters M, Hutchinson AB. The Effectiveness of HIV Partner Counseling and Referral Services in Increasing Identification of HIV-Positive Individuals: A Systematic Review. American Journal of Preventive Medicine. 2007;33(2, Supplement):S89-S100. doi: <https://doi.org/10.1016/j.amepre.2007.04.015>.

6. Kamanga G, Brown L, Jawati P, Chiwanda D, Nyirenda N. Maximizing HIV partner notification opportunities for index patients and their sexual partners in Malawi. Malawi Med J. 2015;27(4):140-4. Epub 2016/03/10. PubMed PMID: 26955435; PubMed Central PMCID: PMCPMC4761705.

7. Wamuti BM, Erdman LK, Cherutich P, Golden M, Dunbar M, Bukusi D, et al. Assisted partner notification services to augment HIV testing and linkage to care in Kenya: study protocol for a cluster randomized trial. Implementation Science. 2015;10(1):23. doi: 10.1186/s13012-015-0212-6.

8. Dovel K, Yeatman S, Watkins S, Poulin M. Men’s heightened risk of AIDS-related death: the legacy of gendered HIV testing and treatment strategies. AIDS (London, England). 2015;29(10):1123.

9. Siu GE, Wight D, Seeley JA. Masculinity, social context and HIV testing: an ethnographic study of men in Busia district, rural eastern Uganda. BMC Public Health. 2014;14(1):33. doi: 10.1186/1471-2458-14-33.

10. Skovdal M, Campbell C, Madanhire C, Mupambireyi Z, Nyamukapa C, Gregson S. Masculinity as a barrier to men's use of HIV services in Zimbabwe. Globalization and health. 2011;7(1):1-14.

11. Choko AT, MacPherson P, Webb EL, Willey BA, Feasy H, Sambakunsi R, et al. Uptake, Accuracy, Safety, and Linkage into Care over Two Years of Promoting Annual Self-Testing for HIV in Blantyre, Malawi: A Community-Based Prospective Study. PLOS Medicine. 2015;12(9):e1001873. doi: 10.1371/journal.pmed.1001873.

12. Kumwenda M, Munthali A, Phiri M, Mwale D, Gutteberg T, MacPherson E, et al. Factors Shaping Initial Decision-Making to Self-test Amongst Cohabiting Couples in Urban Blantyre, Malawi. AIDS and Behavior. 2014;18(4):396-404. doi: 10.1007/s10461-014-0817-9.

13. Wood BR, Ballenger C, Stekler JD. Arguments for and against HIV self-testing. HIV/AIDS - Research and Palliative Care. 2014;6:117-26. doi: 10.2147/HIV.S49083.

14. Choko AT, Desmond N, Webb EL, Chavula K, Napierala-Mavedzenge S, Gaydos CA, et al. The uptake and accuracy of oral kits for HIV self-testing in high HIV prevalence setting: a cross-sectional feasibility study in Blantyre, Malawi. PLoS Med. 2011;8(10):e1001102. Epub 2011/10/13. doi: 10.1371/journal.pmed.1001102. PubMed PMID: 21990966; PubMed Central PMCID: PMCPMC3186813.

15. MacPherson P, Lalloo DG, Webb EL, Maheswaran H, Choko AT, Makombe SD, et al. Effect of Optional Home Initiation of HIV Care Following HIV Self-testing on Antiretroviral Therapy Initiation Among Adults in Malawi: A Randomized Clinical Trial. JAMA. 2014;312(4):372-9. doi: 10.1001/jama.2014.6493.

16. Maheswaran H, Petrou S, MacPherson P, Choko AT, Kumwenda F, Lalloo DG, et al. Cost and quality of life analysis of HIV self-testing and facility-based HIV testing and counselling in Blantyre, Malawi. BMC Medicine. 2016;14(1):34. doi: 10.1186/s12916-016-0577-7.

17. Asiimwe S, Oloya J, Song X, Whalen CC. Accuracy of Un-supervised Versus Provider-Supervised Self-administered HIV Testing in Uganda: A Randomized Implementation Trial. AIDS and Behavior. 2014;18(12):2477-84. doi: 10.1007/s10461-014-0765-4.

18. Ng OT, Chow AL, Lee VJ, Chen MI, Win MK, Tan HH, et al. Accuracy and user-acceptability of HIV self-testing using an oral fluid-based HIV rapid test. PLoS One. 2012;7(9):e45168. Epub 2012/10/03. doi: 10.1371/journal.pone.0045168. PubMed PMID: 23028822; PubMed Central PMCID: PMCPMC3444491 receipt of GlaxoSmithKline funding for unrelated research. There are no patents, products in development or marketed products to declare. This does not alter the authors' adherence to all the PLoS ONE policies on sharing data and materials, as detailed online in the guide for authors.

19. Zachary D, Mwenge L, Muyoyeta M, Shanaube K, Schaap A, Bond V, et al. Field comparison of OraQuick® ADVANCE Rapid HIV-1/2 antibody test and two blood-based rapid HIV antibody tests in Zambia. BMC Infectious Diseases. 2012;12(1):183. doi: 10.1186/1471-2334-12-183.

20. Long L, Brennan A, Fox MP, Ndibongo B, Jaffray I, Sanne I, et al. Treatment outcomes and cost-effectiveness of shifting management of stable ART patients to nurses in South Africa: an observational cohort. PLoS medicine. 2011;8(7):e1001055. doi: 10.1371/journal.pmed.1001055. PubMed PMID: 21811402.

21. Rosen S, Long L, Sanne I. The outcomes and outpatient costs of different models of antiretroviral treatment delivery in South Africa. Tropical Medicine & International Health. 2008;13(8):1005-15. doi: <https://doi.org/10.1111/j.1365-3156.2008.02114.x>.

22. Scott CA, Iyer H, Bwalya DL, McCoy K, Meyer-Rath G, Moyo C, et al. Retention in care and outpatient costs for children receiving antiretroviral therapy in Zambia: a retrospective cohort analysis. PLoS One. 2013;8(6):e67910. Epub 2013/07/11. doi: 10.1371/journal.pone.0067910. PubMed PMID: 23840788; PubMed Central PMCID: PMCPMC3695874.
