# Supplementary material for: Effect of index HIV self-testing for sexual partners of clients enrolled in antiretroviral therapy (ART) programs in Malawi: A randomized controlled trial
Source: PLoS Med. 2023 Aug 4;20(8):e1004270. doi: 10.1371/journal.pmed.1004270 (PMC10403056; doi:10.1371/journal.pmed.1004270)
Supplement: S1 Table — (DOCX) [file pmed.1004270.s003.docx]

| **S1 Table: Sensitivity analyses using true intention-to-treat, reported by index (ART) clients (n=484)** | | | | | |  | |  | |
| --- | --- | --- | --- | --- | --- | --- | --- | --- | --- |
| HIV Testing Service | PRS Arm | HIVST Arm | RR  (95% CI) | p-value | aRR  (95% CI)^ | | p-value | |  |
|  | n(%) | n(%) |  |  |  |  |  |  |  |
| Index client delivered intervention | 98/135 (72.6%) | 231/349 (66.2%) | 0.91  (0.85, 0.98) | 0.007 | 0.97  (0.90, 1.04) | | 0.414 | |  |
| Female index partner | 73/105 (69.5%) | 179/266 (67.3%) | 0.98  (0.89, 1.05) | 0.424 | 0.99  (0.88, 1.11) | | 0.829 | |  |
| Male index partner | 25/30 (83.3%) | 52/83 (62.7%) | 0.75  (0.66, 0.85) | <0.001 | 0.93  (0.90, 0.96) | | <0.001 | |  |
| Index partner tested | 27/135 (20.0%) | 183/349 (52.4%) | 2.62  (2.55, 2.70) | <0.001 | 2.77  (2.56, 3.00) | | <0.001 | |  |
| Female index partner | 17/105 (16.2%) | 135/266 (50.8%) | 3.13  (2.87, 3.43) | <0.001 | 3.19  (2.68, 3.81) | | <0.001 | |  |
| Male index partner | 10/30 (33.3%) | 48/83 (57.8%) | 1.73  (1.44, 2.10) | <0.001 | 2.38  (1.86, 3.06) | | <0.001 | |  |
| Index partner tested HIV-positive | 4/135 (2.9%) | 30/349 (8.6%) | 2.90  (1.09, 7.79) | 0.032 | 2.94  (0.94, 9.17) | | 0.063 | |  |
| Female index partner | 4/105 (3.8%) | 28/266 (10.5%) | 2.76  (1.08, 7.06) | 0.034 | 2.72  (0.96, 7.72) | | 0.061 | |  |
| Male index partner | 0/30 (0.0%) | 2/83 (2.4%) | - | - | - | | - | |  |
| HIV+ index partner-initiated ART at 12-months | 3/135 (2.2%) | 14/349 (4.0%) | - | - | - | | - | |  |
| Female index partner | 3/105 (2.9%) | 14/266 (5.3%) | - | - | - | | - | |  |
| Male index partner | 0/30 (0.0%) | 0/83 (0.0%) | - | - | - | | - | |  |
| *ART, antiretroviral therapy; PRS, partner referral slip; HIVST, HIV self-test; RR, rate ratio; aRR, adjusted rate ratio; CI, confidence interval*  ^adjusted for age and marital status | | | | | | | | |  |
|  |  |  |  |  |  | |  | |  |
