# Supplementary material for: Effect of index HIV self-testing for sexual partners of clients enrolled in antiretroviral therapy (ART) programs in Malawi: A randomized controlled trial
Source: PLoS Med. 2023 Aug 4;20(8):e1004270. doi: 10.1371/journal.pmed.1004270 (PMC10403056; doi:10.1371/journal.pmed.1004270)
Supplement: S3 Table — (DOCX) [file pmed.1004270.s005.docx]

| **S3 Table: Baseline characteristics among those with a primary outcome, reported by index client (n=364)** | | | | |  |  |
| --- | --- | --- | --- | --- | --- | --- |
| **Respondent Characteristic** | **Total** | **PRS Arm** | **HIVST Arm** | **p-value** |  |  |
|  | n (%) | n (%) | n (%) |  |  |  |
| n | 364 | 107 (29.4%) | 257 (70.6%) |  |  |  |
| **Client Information** |  |  |  |  |  |  |
| **Demographic variables** |  |  |  |  |  |  |
| Male | 80 (22.0%) | 26 (24.3%) | 54 (21.0%) | 0.476 |  |  |
| Mean age, years (SD) | 37 (11.6) | 37 (12.4) | 37 (11.2) | 0.854 |  |  |
| Median years of education, (IQR) | 4 (1-8) | 4 (1-8) | 5 (1-8) | 0.706 |  |  |
| Relationship type with primary partner |  |  |  |  |  |  |
| Married/living together | 302 (82.9%) | 88 (82.2%) | 214 (83.3%) |  |  |  |
| Steady partner | 44 (12.1%) | 16 (15.0%) | 28 (10.9%) |  |  |  |
| New/Infrequent partner | 18 (5.0%) | 3 (2.8%) | 15 (5.8%) | 0.324 |  |  |
| Median years in a relationship, (IQR) | 5.5 (2-15) | 4 (2-14) | 6 (2-15) | 0.640 |  |  |
| Household wealth quantiles |  |  |  |  |  |  |
| Low quantile | 122 (33.5%) | 41 (38.3%) | 81 (31.5%) |  |  |  |
| Middle quantile | 121 (33.2%) | 29 (27.1%) | 92 (35.8%) |  |  |  |
| High quantile | 121 (33.3%) | 37 (34.6%) | 84 (32.7%) | 0.241 |  |  |
| Worked for pay in last month | 192 (52.8%) | 55 (51.4%) | 137 (53.3%) | 0.740 |  |  |
| Median number of living children, (IQR) | 3 (2-5) | 3 (2-5) | 3 (2-5) | 0.141 |  |  |
| Has living child with the primary partner | 227 (64.3%) | 63 (61.2%) | 164 (65.6%) | 0.429 |  |  |
| **HIV services** |  |  |  |  |  |  |
| Median years on ART, (IQR) | 4.3 (1-8) | 4.2 (1-9) | 4.3 (2-7) | 0.716 |  |  |
| Disclosed HIV status to a non-sexual partner | 324 (89.0%) | 96 (89.7%) | 228 (88.7%) | 0.428 |  |  |
| Have a close friend on ART (talk with them at least 1/week) | 170 (54.3%) | 51 (53.7%) | 119 (54.6%) | 0.883 |  |  |
| More than one sexual partner in past 12  months | 29 (7.9%) | 10 (9.4%) | 19 (7.4%) | 0.531 |  |  |
| **Primary Partner Information** |  |  |  |  |  |  |
| **Demographic variables** |  |  |  |  |  |  |
| Male | 284 (78.0%) | 81 (75.7%) | 203 (79.0%) | 0.490 |  |  |
| Mean age, years (SD) | 40 (11.5) | 40 (12.5) | 40 (11.1) | 0.643 |  |  |
| **Relationship with primary partner** |  |  |  |  |  |  |
| Disclosed HIV status to primary partner | 325 (92.1%) | 93 (90.3%) | 232 (92.8%) | 0.428 |  |  |
| Talk with partner at least once per week | 338 (95.8%) | 98 (95.2%) | 240 (96.0%) | 0.457 |  |  |
| Confident they would still be with this  partner in 12 months | 291 (82.4%) | 82 (79.6%) | 209 (83.6%) | 0.371 |  |  |
| **Region** |  |  |  |  |  |  |
| Southern | 229 (62.9%) | 72 (67.3%) | 157 (61.1%) |  |  |  |
| Central | 135 (37.1%) | 35 (32.7%) | 100 (38.9%) | 0.265 |  |  |
| *ART, antiretroviral therapy; PRS, partner referral slip; HIVST, HIV self-test; SD, standard deviation, IQR, interquartile range*  ^adjusted for age and marital status | | | | | | |
