# Supplementary material for: Effect of index HIV self-testing for sexual partners of clients enrolled in antiretroviral therapy (ART) programs in Malawi: A randomized controlled trial
Source: PLoS Med. 2023 Aug 4;20(8):e1004270. doi: 10.1371/journal.pmed.1004270 (PMC10403056; doi:10.1371/journal.pmed.1004270)
Supplement: S1 Fig — (DOCX) [file pmed.1004270.s006.docx]

**S1 Fig: Kaplan Meier Curve showing time to ART initiation among HIVST users who test HIV+**

**(n=30)**

| *ART, antiretroviral therapy; HIVST, HIV self-test* |
| --- |
